# Mapping the association between back pain and type 2 diabetes: A cross-sectional and longitudinal study of adult Spanish twins

**DOI:** 10.1371/journal.pone.0174757

**Published:** 2017-04-03

**Authors:** Amabile Dario, Manuela Ferreira, Kathryn Refshauge, Alison Harmer, Juan Sánchez-Romera, Francisco Pérez-Riquelme, Ligia Cisneros, Juan Ordoñana, Paulo Ferreira

**Affiliations:** 1 Faculty of Health Sciences, The University of Sydney, Sydney, NSW, Australia; 2 The George Institute for Global Health, Sydney Medical School, The University of Sydney, Sydney, NSW, Australia; 3 Institute of Bone and Joint Research, Sydney Medical School, The University of Sydney, Sydney, NSW, Australia; 4 Murcia Twin Registry, Department of Human Anatomy and Psychobiology, University of Murcia, Murcia, Spain; 5 Biomedical Research Institute of Murcia (IMIB-Arrixaca-UMU), University Clinical Hospital “Virgen de la Arrixaca”, Murcia, Spain; 6 Department of Physiotherapy, Federal University of Minas Gerais, Belo Horizonte, Minas Gerais, Brazil; Rush University Medical Center, UNITED STATES

## Abstract

**Background:**

Back pain and type 2 diabetes often co-occur, resulting in greater impact on people’s health and complexity in their care. Plausible causal mechanisms for this association have been proposed, yet the nature of the link remains unclear. We therefore explored the direction of the association between type 2 diabetes and chronic back pain in twins, controlling for genetics and early environmental confounding.

**Methods:**

2,096 and 1,098 twins were included in the cross-sectional and longitudinal analyses, respectively. Any or severe (≥ 9) low back pain (LBP), neck pain (NP), and spinal pain (concurrent LBP and NP) and type 2 diabetes were investigated. Sequential analyses were performed using logistic regression. Firstly, twins were analysed unpaired (adjusted age and gender): *total sample analyses*. Then, to control for genetic and shared environmental factors, a *co-twin case-control analysis* was performed including monozygotic and dizygotic twin pairs discordant for back pain (cross-sectional only).

**Results:**

In the cross-sectional total sample analyses, type 2 diabetes was associated with chronic spinal pain (OR 1.61; 95%CI 1.12 to 2.31), severe chronic spinal pain (OR 3.33; 95%CI 1.47 to 7.53), chronic NP (OR 1.37; 95%CI 1.01 to 1.85), severe chronic NP (OR 2.28; 95%CI 1.24 to 4.21), and severe chronic LBP (OR 1.63; 95%CI 1.00 to 2.64). After further adjustment for genetic and shared environmental factors, none of the associations remained significant. The longitudinal analyses indicated that the presence of type 2 diabetes did not increase the risk of future back pain, or vice-versa, after two to four years.

**Conclusions:**

Chronic back pain (spinal pain, NP, or LBP) was associated with the prevalence of type 2 diabetes. Associations are stronger for severe cases of pain. Future research should investigate the temporal relationships between these conditions with longer follow up in twins.

## Introduction

Diabetes, low back pain (LBP) and neck pain (NP) are all recognized major public health problems [1–3]. They are common and costly conditions, ranking among the top seven causes of years lived with disability worldwide [1]. Recent studies have reported that diabetes commonly coexists with LBP and NP [4–7]. The prevalence of LBP among people with diabetes is twice as high as among age- and gender-matched controls [8]. Importantly, patients with concurrent diabetes and LBP have more frequent recurrence of pain, higher levels of LBP-specific disability, and poorer general health than those with LBP in isolation [6, 9]. Furthermore, those presenting with both conditions are twice as likely to be admitted to hospital [OR: 2.02; 95% confidence interval (CI): 1.69 to 2.40] and to have surgery for cervical or lumbar disc disease, which incurs significant health care expenditure [7, 9–11].

Recent evidence suggests that diabetes and back pain, including LBP, NP and spinal pain (concurrent LBP and NP), not only co-exist, but may in fact be bi-directionally linked. The hyperglycemia and altered fat metabolism commonly present in diabetes have been linked to pathoanatomical changes of the spine, such as early degeneration of vertebrae, cartilage, and intervertebral discs [12–16]. These changes are a frequent finding in osteoarthritic spinal joints and have been associated with pain [17–20]. Conversely, chronic pain is well known to have an adverse impact on health behaviours such as physical activity and diet, and these lifestyle choices may induce type 2 diabetes [21–23].

Apart from a possible bi-directional relationship between diabetes and back pain, it is also plausible that these health conditions coexist due to common risk factors, such as genetic influences, on their pathogeneses [24]. Family-based studies have consistently suggested the presence of a major genetic component underlying the variation (heritability) of LBP (52%; 95% CI 33 to 72%), NP (48%; 95% CI 29 to 67%) and type 2 diabetes (72%; 95% CI 61 to 78%) [25, 26]. Moreover, evidence from twin studies indicated that genetics may be a confounder in the association between disc degeneration and diabetes in twin [24, 27]. Therefore, genetics should be taken into account to obtain more precise estimates when investigating a possible direct path between back pain and diabetes.

Considering that the global population is ageing and becoming more obese, a future increase in the burden of diabetes and back pain is likely to occur, as these conditions are commonly observed in the older and obese populations [28–30]. Understanding potential causal risk factors for diabetes and back pain is therefore paramount for optimizing treatment and prevention of these conditions. Thus, we explored the bi-directional association, in terms of precision and magnitude, between type 2 diabetes and chronic back pain [chronic LBP, chronic NP, and chronic spinal pain] using a Spanish twin sample. The use of twin pairs discordant for a health condition, in this case back pain, allows the influence of critical potential confounders such as genetic and early environmental factors to be controlled.

## Method

### Study design

Cross-sectional and longitudinal observational study with a co-twin case-control design.

### Participants and data collection

The study sample comprised adult twins from the Murcia Twin Registry (MTR) in Spain who were born between 1940 and 1966 in the region of Murcia. For the present study, twins recruited by the MTR in the second data wave (baseline: 2009 to 2011) and third data wave (follow-up: 2013) who provided information on diabetes and back pain were included. Data were collected face-to-face or via telephone interviews conducted by research assistants who were blinded to the predictors and outcomes of the study. All recruitment and data collection procedures for the MTR were approved by the University of Murcia Research Ethics Committee (31). Initially, participants were contacted through postal letter to explain the objectives of the MTR and invite the twins for collaboration. When a telephone interview was conducted, oral informed consent was obtained prior to any data collection. When interviewed in person, participants provided written informed consent.

### Zygosity evaluation

Twin zygosity was assessed by DNA in 338 pairs and by a 12-item questionnaire in the remainder of the sample. The questionnaire, which identifies the degree of similarity among twin pairs, is in agreement with DNA testing in approximately 96% of cases [31].

### Assessment of LBP, NP, and spinal pain

Information on chronic LBP and chronic NP was drawn from self-reported responses derived from the Spanish National Health Survey [32]. At baseline, participants were asked “Have you ever suffered from chronic LBP (or NP)?”, followed by, in case of an affirmative answer “Has it been diagnosed by a doctor?”. The definition of chronic back pain utilised in the survey was the presence of pain in the lower back or neck area lasting for six months or longer, including seasonal or recurrent episodes, regardless of the origin of pain (e.g. degeneration, trauma, unknown). Participants were fully apprised of this definition during the process of data collection. Participants answering “yes” to either or both the LBP or NP questions were categorized as having chronic LBP alone, chronic NP alone, or chronic spinal pain (concurrent LBP and NP). At follow-up, participants who answered positively to the same chronic LBP and chronic NP questions that were administered at baseline were asked additional questions to gather information on pain intensity: “How intense was your pain in the last episode (0 = no pain at all; 10 = the worst pain ever)?”.

### Assessment of type 2 diabetes

Similar to the assessment of back pain, information on diabetes was assessed using self-reported responses to the Spanish National Health Survey [32]. Participants were asked the following questions “Have you ever suffered diabetes?” and when the answer was affirmative, “Has it been diagnosed by a doctor?”. An additional question was asked about medication: “Did you take medication for diabetes in the previous month?” Initially, those who answered “yes” to one or more of these questions were categorized as having diabetes. This information was then linked to the regional databases of the Murcia Health Council, which include data about virtually all patients using the public health system in the geographical area of the MTR. Only participants for whom the diagnosis of type 2 diabetes could be confirmed (diagnosed by a physician) were considered as cases. Participants with type 1 diabetes or non-confirmed self-reported type 2 diabetes were excluded (0.5% and 1.9% of the total sample, respectively).

### Assessment of covariates

Potential confounders were selected based on plausible associations with back pain and diabetes, as well as data availability. Variables investigated included age, sex, body mass index (BMI), engagement in physical activity (work-related and leisure), and smoking history. To assess engagement in physical activity and smoking history we used categorical self-reported responses to the Spanish National Health Survey [32]. BMI was calculated from self-reported height and weight, and used as a continuous variable. For smoking history, participants were categorized as current smoker or never smoker/ex-smoker. For leisure-time physical activity, participants were categorized as sedentary (no engagement in recreational physical activity) or regularly physically active (low/moderate/vigorous physical activity engagement). For work-related physical activity, participants were categorized as sedentary (low/no engagement in work related physical activity such as mainly sitting or light physical efforts) or as doing tasks that require a strong physical effort (moderate/vigorous physical activity engagement).

### Statistical analysis

**Fig 1:**
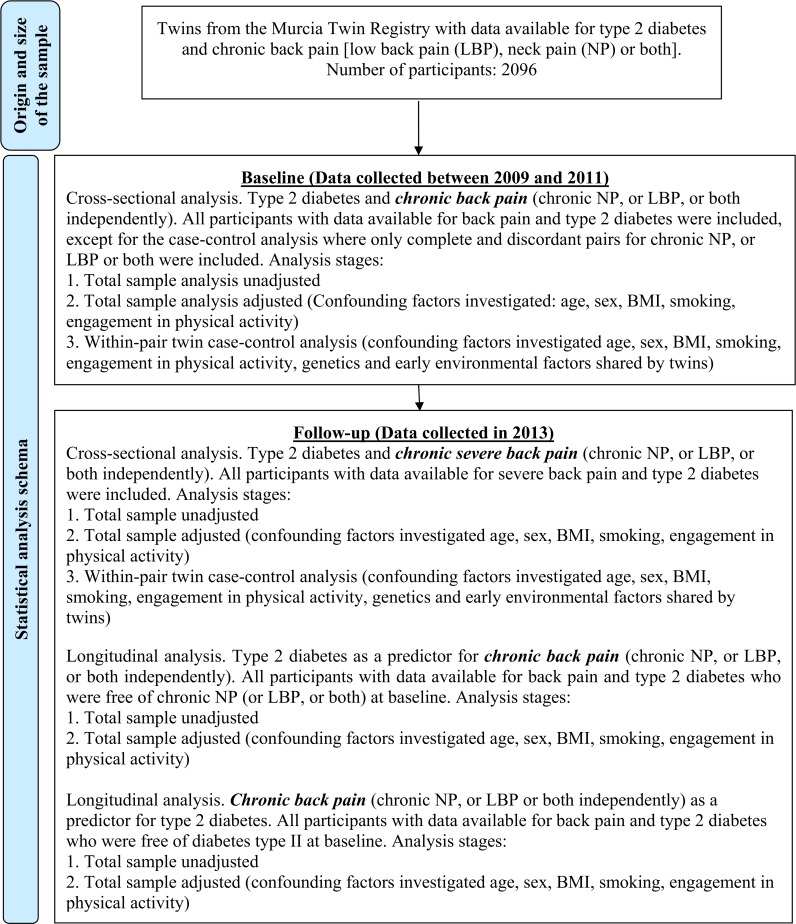
Statistical analysis schema and sample size. The level of adjustment for confounding factors increases throughout the analytical stages.

Descriptive analyses were performed on demographic and clinical characteristics of the cohort at baseline and follow-up. We then investigated the cross-sectional and longitudinal associations between diabetes and back pain using univariate and multivariable regression models. To adjust the models for the similarities shared by twins (i.e. to control for data dependence due to twin sample), we used a robust sandwich estimator (*cluster* command in STATA) in the total sample analyses. In the multivariable regression analyses, we adjusted the total sample models by age and sex to ensure comparability with the co-twin case-control models. Possible additional confounders (BMI, engagement in physical activity, and smoking history) were only included in the models when the p-values were <0.2 for the associations with both the outcome and exposure. Fig 1 describes the statistical analysis schema.

#### Cross-sectional analyses

To explore a possible association between type 2 diabetes and spinal pain, NP or LBP, we conducted cross-sectional analyses using the baseline data. In addition, with the availability of data on pain intensity collected at the follow-up assessment (2013), we investigated the potential association between type 2 diabetes and severe cases of spinal pain (or NP, or LBP). Participants in the upper quartile of the distribution of the pain intensity variable, reporting pain equal to or higher than 9/10, were classified as having severe pain. To control for the possible effects of genetic and early shared environmental factors on the relationship between type 2 diabetes and chronic spinal pain (or NP, or LBP), we conducted a co-twin case-control analysis. Only complete and discordant twin pairs (i.e. one twin reported chronic spinal pain, whereas the co-twin did not) for each back pain outcome were included. Theoretically, the co-twin control design enables adjustment of the estimates for a large number of confounding factors that twins share, including genetics, as monozygotic and dizygotic twin pairs share approximately 100% and 50% of their genes, respectively. Furthermore, twins tend to be exposed to a common environment until early adulthood.

#### Longitudinal analysis

(i) To investigate type 2 diabetes as a risk factor for chronic spinal pain (or NP, or LBP), twins were included when they did not report chronic spinal pain (or NP, or LBP) at baseline and had complete data available at both baseline and follow-up. A similar method was used to investigate whether type 2 diabetes increased the risk of severe cases of chronic spinal pain (or NP, or LBP) with twins being included only if they did not report severe spinal pain (or NP, or LBP) at baseline. (ii) To investigate chronic spinal pain (or NP, or LBP) as risk factors for type 2 diabetes, twins were included when they did not report diabetes at baseline and had complete data available at both baseline and follow-up.

## Results

### Sample characteristics

At baseline, a total of 2,096 twins were included in our cross-sectional analysis. The mean age of twins was 53.6 [standard deviation (SD) 7.3] years, and the majority of the sample was composed of female twins (55%). On average, twins were overweight (BMI 27.4kg/m^2^, SD 4.5), with 18.8% reporting engagement in work-related physical activity and 54.2% in leisure-time physical activity. The prevalence of chronic spinal pain, NP, and LBP was 18.2% (95% CI 16.5 to 19.8), 28.4% (95% CI 26.5 to 30.3), and 32.2% (95% CI 30.2 to 34.2) respectively, while 10.9% (95% CI 9.6 to 12.3) of twins had a confirmed diagnosis of type 2 diabetes (Table 1).

**Table 1:** Characteristics of study sample, including anthropometric data, lifestyle factors, and type 2 diabetes and back pain status at baseline and follow-up.

Variables	Baseline		Follow-up	
	Mean(SD) or %	n	Mean (SD) or %	n
Age (years)	53.6(7.3)	2096	56.7(7.1)	1613
Height (m)	1.63(1.0)	2049	1.64(9.2)	1502
Weight (kg)	73.2(14.1)	2082	73.2(13.7)	1575
Body mass index (kg/m^2^)	27.4(4.5)	2041	27.2(4.3)	1491
Male	44.8%	940	44.9%	725
Current smokers	36.2%	759	30.8%	496
Work-related physical activity ^a^	18.8%	393	20.8%	336
Leisure-time physical activity ^a^	54.2%	1137	66.0%	1064
Type 2 diabetes^b^	10.9%	229	13.0%	210
Low back pain^b^	32.2%	675	36.8%	593
Neck pain^b^	28.4%	595	24.8%	400
Spinal pain^^b^	18.2%	381	14.8%	239

^ Spinal pain: concurrent lower back and neck pain; SD: standard deviation; n: number of participants

^a^ Percentage engaged in physical activity within group

^b^ Prevalence.

At follow-up, 1613 twins from the original sample had complete data for back pain and type 2 diabetes. The mean age of the twins was 56.7 (SD 7.1) years and their mean BMI was classified as overweight (BMI 27.2 kg/m^2^; SD 4.3). The proportion of those engaged in work-related and leisure-time physical activity were 20.8% and 66.0%, respectively. The prevalence of chronic spinal pain, NP, and LBP was 14.8% (95% CI 13.1 to 16.6), 24.8% (95% CI 22.8 to 27.0), and 36.8% (95% CI 34.5 to 39.2); with 13% (95% CI 11.4 to 14.7) of twins presenting with type 2 diabetes (Table 1). The proportions of incident cases of chronic spinal pain, NP, and LBP at follow-up were 9.0% (95% CI 7.5 to 10.6), 14.7% (95% CI 12.7 to 16.8), and 22.3% (95% CI 19.8 to 24.8), respectively. Incident cases of type 2 diabetes comprised 2.4% (95% CI 1.6 to 3.3) of the twins over two to four years follow-up (Table 1).

### Type 2 diabetes and chronic spinal pain (both LBP and NP)

The analyses, including cross-sectional data from the total sample, demonstrated that type 2 diabetes was associated with chronic spinal pain [unadjusted odds ratio (OR) 1.49; 95% CI 1.07 to 2.09; adjusted OR 1.61; 95% CI 1.12 to 2.31] and severe chronic spinal pain (unadjusted OR 2.94; 95% CI 1.44 to 5.99; adjusted OR 3.33; 95% CI 1.47 to 7.53) (Table 2). When the analyses were separated by sex, type 2 diabetes was associated with chronic spinal pain in females only (unadjusted OR 1.76; 95% CI 1.17 to 2.66; adjusted OR 1.64; 95% CI 1.06 to 2.53). However, for severe chronic spinal pain, type 2 diabetes was strongly associated among both females (unadjusted OR 2.71; 95% CI 1.03 to 7.11), and males (unadjusted OR 3.46; 95% CI 1.13 to 10.59; adjusted OR 4.80; 95% CI 1.37 to 16.84). After adjusting for genetic and shared environmental factors using 201 and 26 twin pairs discordant for chronic spinal pain and severe chronic spinal pain respectively, the magnitude of association reduced and was no longer significant.

**Table 2:** Association between type 2 diabetes and chronic spinal pain (concurrent low back pain and neck pain) for all participants and by sex.

Models	All participants		Female		Male	
	OR (95% CI)	n	OR (95% CI)	n	OR (95% CI)	n
**Cross-sectional analysis**						
*Chronic SP*						
Unadjusted	**1.49 (1.07 to 2.09)**	2096	**1.76 (1.17 to 2.66)**	1156	1.27 (0.65 to 2.47)	940
Adjusted^1^^,^ ^2^^,^^3^	**1.61 (1.12 to 2.31)**	2084	**1.64 (1.06 to 2.53)**	1153	1.69 (0.81 to 3.49)	931
MZ and DZ pairs^2^^,^^3^*	1.12 (0.58 to 2.15)	402	1.07 (0.43 to 2.64)	222	2.04 (0.37 to 11.26)	76
*Severe Chronic SP*^*#*^						
Unadjusted	**2.94 (1.44 to 5.99)**	1485	**2.71 (1.03 to 7.11)**	813	**3.46 (1.13 to 10.59)**	672
Adjusted ^1^	**3.33 (1.47 to 7.53)**	1485	2.60 (0.87 to 7.82)	813	**4.80 (1.37 to 16.84)**	672
MZ and DZ pairs*	2.90 (0.48 to 17.42)	52	-	-	-	-
**Longitudinal analysis:** Type 2 diabetes as a risk factor for SP						
*Chronic SP*						
Unadjusted	0.95 (0.49 to 1.84)	1293	0.78 (0.30 to 2.05)	637	1.16 (0.46 to 2.92)	656
Adjusted ^1^^,^^2^	0.85 (0.42 to 1.73)	1284	0.60 (0.20 to 1.81)	636	1.16 (0.44 to 3.05)	648
*Severe Chronic SP*^*#*^						
Unadjusted	3.80 (0.91 to 15.82)	98	7.17 (0.89 to 57.47)	52	2.20 (0.31 to 15.49)	46
Adjusted ^1^^,^^2^	3.67 (0.84 to 16.03)	98	7.16 (0.91 to 56.41)	52	2.24 (0.26 to 19.47)	46
**Longitudinal analysis:** SP as a risk factor for type 2 diabetes						
*Type 2 diabetes*						
Unadjusted	0.80 (0.31 to 2.11)	1399	1.63 (0.54 to 4.93)	778	-	-
Adjusted^1^	1.01 (0.39 to 2.59)	1399	1.52 (0.50 to 4.65)	778	-	-

SP: Spinal pain; OR: Odds ratio; CI: Confidence interval; MZ: Monozygotic; DZ: Dizygotic; n: Number of participants in each analysis stage

^1^ Adjusted for age and sex

^2^ Adjusted for work-related physical activity

^3^ Adjusted for body mass index

^#^ Severe pain: pain ≥ 9 on visual analogical scale (0 to 10) in the last episode

* Case-control analysis: twins are discordant for spinal pain status. Analyses stratified by gender only include same-sex pairs. Numbers in **bold** represent statistically significant results (p≤ 0.05).

The longitudinal analysis for the total sample showed no association between type 2 diabetes and risk of developing severe chronic spinal pain after two to four years follow-up. Likewise, presence of chronic spinal pain did not increase the risk of future type 2 diabetes.

### Type 2 diabetes and chronic NP

The cross-sectional analysis of the total sample demonstrated that type 2 diabetes was associated with chronic NP (unadjusted OR 1.35; 95% CI 1.02 to 1.79; adjusted OR 1.37; 95% CI 1.01 to 1.85) (Table 3). When the analysis was separated by sex, type 2 diabetes was only associated with chronic NP in females (unadjusted OR 1.72; 95% CI 1.18 to 2.51; adjusted OR 1.58; 95% CI 1.07 to 2.34). The positive associations in the total and female only samples did not remain significant after adjustment for genetic and shared environmental factors using 276 and 139 twin pairs discordant for chronic NP, respectively.

**Table 3:** Association between type 2 diabetes and chronic neck pain for all participants and by sex.

Models	All participants		Female		Male	
	OR (95% CI)	n	OR (95% CI)	n	OR (95% CI)	n
**Cross-sectional analysis**						
*Chronic NP*						
Unadjusted	**1.35 (1.02 to 1.79)**	2096	**1.72 (1.18 to 2.51)**	1156	1.04 (0.60 to 1.79)	940
Adjusted ^1^^,^^2^^,^^3^^,^^4^	**1.37 (1.01 to 1.85)**	2074	**1.58 (1.07 to 2.34)**	1146	1.09 (0.61 to 1.95)	928
MZ and DZ pairs^2^^,^^3^^,^^4^*	1.23 (0.62 to 2.27)	552	1.69 (0.66 to 4.32)	278	0.85 (0.23 to 3.19)	118
*Severe Chronic NP*^#^						
Unadjusted	**2.11 (1.17 to 3.79)**	1511	**2.29 (1.04 to 5.04)**	826	1.95 (0.80 to 4.77)	685
Adjusted ^1^	**2.28 (1.24 to 4.21)**	1511	2.19 (0.99 to 4.87)	826	2.44 (0.92 to 6.48)	685
MZ and DZ pairs*	0.91 (0.30 to 2.78)	86	1.00 (0.06 to 15.99)	30	1.33 (0.29 to 5.96)	30
**Longitudinal analysis:** Type 2 diabetes as a risk factor for NP						
*Chronic NP*						
Unadjusted	1.29 (0.77 to 2.18)	1126	1.27 (0.56 to 2.89)	519	1.31 (0.66 to 2.61)	607
Adjusted ^1^^,^^2^^,^^4^	1.16 (0.65 to 1.91)	1111	0.82 (0.34 to 2.01)	513	1.38 (0.68 to 2.82)	598
*Severe Chronic NP*^#^						
Unadjusted	1.88 (0.58 to 6.11)	138	5.33 (0.92 to 31.06)	63	0.87 (0.16 to 4.72)	75
Adjusted ^#^^1^^,^^2^	1.91 (0.52 to 6.95)	138	6.42 (0.92 to 44.53)	63	0.74 (0.12 to 4.64)	75
**Longitudinal analysis:** NP as a risk factor for type 2 diabetes						
*Type 2 diabetes*						
Unadjusted	0.80 (0.31 to 2.11)	1399	1.63 (0.54 to 4.93)	778	-	-
Adjusted^1^	1.01 (0.39 to 2.59)	1399	1.52 (0.50 to 4.65)	778	-	-

NP: Neck Pain; OR: Odds ratio; CI: Confidence interval; MZ: Monozygotic; DZ: Dizygotic; n: Number of participants in each analysis stage

^1^Adjusted for age and sex

^2^Adjusted for work-related physical activity

^3^Adjusted for body mass index

^4^Adjusted for smoking

^#^ Severe pain: pain ≥ 9 on visual analogical scale (0 to 10) in the last episode

* Case-control analysis: twins are discordant for neck pain status. Analyses stratified by gender only include same-sex pairs. Numbers in **bold** represent statistically significant results (p≤ 0.05).

We also found that type 2 diabetes was strongly associated with higher prevalence of severe chronic NP (unadjusted OR 2.11; 95% CI 1.17 to 3.79; adjusted OR 2.28; 95% CI 1.24 to 4.21). When the analyses were separated by sex, type 2 diabetes was associated with severe chronic NP in females only in the unadjusted analysis (OR 2.28; 95% CI 1.04 to 5.04) and was close to statistical significance in the adjusted analysis (OR 2.19; 95% CI 0.99 to 4.87). The positive associations for severe chronic NP in the total and female only samples did not remain after adjustment for genetic and shared environmental factors in 43 and 15 discordant twin pairs, respectively. In the total sample analysis using longitudinal data, type 2 diabetes did not increase the risk of developing chronic NP, or vice-versa, after two to four years.

### Type 2 diabetes and chronic LBP

The cross-sectional analysis of the total sample showed that type 2 diabetes was only associated with higher prevalence of severe chronic LBP in the adjusted total sample analysis (OR 1.63; 95% CI 1.00 to 2.64) (Table 4). After adjusting for genetic and shared environmental factors using 73 twin pairs discordant for severe chronic LBP, the association was no longer significant. The longitudinal analyses showed that type 2 diabetes did not increase the risk of developing chronic LBP, or vice-versa, after two to four years.

**Table 4:** Association between type 2 diabetes and chronic low back pain for all participants and by sex.

Models	All participants		Female		Male	
	OR (95% CI)	n	OR (95% CI)	n	OR (95% CI)	n
**Cross-sectional analysis**						
*Chronic LBP*						
Unadjusted	1.07 (0.80 to 1.45)	2096	1.38 (0.93 to 2.04)	1156	0.79 (0.48 to 1.29)	940
Adjusted^1^^,^^2^	1.18 (0.86 to 1.60)	2084	1.35 (0.90 to 2.03)	1153	0.96 (0.57 to 1.62)	931
MZ and DZ pairs*	0.84 (0.45 to 1.55)	610	0.89 (0.34 to 2.30)	284	1.02 (0.25 to 4.10)	136
*Severe Chronic LBP*^#^						
Unadjusted	1.36 (0.86 to 2.15)	1525	1.52 (0.83 to 2.81)	834	1.20 (0.60 to 2.39)	691
Adjusted^1^	**1.63 (1.00 to 2.64)**	1525	1.88 (0.99 to 3.58)	834	1.40 (0.67 to 2.95)	691
MZ and DZ pairs*	2.75 (0.54 to 13.91)	146	-	-	-	-
**Longitudinal analysis:** Type 2 diabetes as a risk factor for LBP						
*Chronic LBP*						
Unadjusted	0.76 (0.47 to 1.22)	1084	0.87 (0.41 to 1.85)	515	0.67 (0.36 to 1.27)	569
Adjusted^1^^,^^2^^,^^3^	0.84 (0.51 to 1.40)	1077	0.73 (0.32 to 1.63)	514	0.98 (0.50 to 1.92)	563
*Severe Chronic LBP*^#^						
Unadjusted	1.88 (0.71 to 5.02)	218	1.25 (0.23 to 6.85)	89	2.38 (0.70 to 8.12)	129
Adjusted ^#^^1^^,^^2^	1.91 (0.67 to 5.46)	218	1.38 (0.24 to 7.99)	89	2.23 (0.56 to 8.77)	129
**Longitudinal analysis:** LBP as a risk factor for type 2 diabetes						
*Type 2 diabetes*						
Unadjusted	0.92 (0.45 to 1.90)	1399	2.03 (0.70 to 5.94)	778	0.43 (0.11 to 1.76)	621
Adjusted^1^	1.10 (0.54 to 2.22)	1399	1.92 (0.66 to 5.60)	778	0.49 (0.12 to 1.97)	621

LBP: Low back pain; OR: Odds ratio; CI: Confidence interval; MZ: Monozygotic; DZ: Dizygotic; n: Number of participants

^1^Adjusted for age and sex

^2^ Adjusted for work-related physical activity

^3^Adjusted for body mass index

^#^ Severe pain: pain ≥ 9 on visual analogical scale (0 to 10) in the last episode

* Case-control analysis: twins are discordant for low back pain status. Analyses stratified by gender only include same-sex pairs. Numbers in **bold** represent statistically significant results (p≤ 0.05).

## Discussion

### Main findings

Our findings suggest a positive association between type 2 diabetes and chronic back pain in the cross-sectional analyses. Those in our cohort with type 2 diabetes were more likely to report chronic low back, neck, and spinal pain. The associations tended to be stronger for *severe* cases of chronic pain (e.g. severe chronic spinal pain: adjusted OR 3.33) than any pain (chronic spinal pain: adjusted OR 1.61). Nevertheless, our findings do not provide strong and conclusive evidence of a causal relationship between type 2 diabetes and back pain. Firstly, none of the associations remained significant after further adjusting for the genetic and early environmental factors shared by twins. Moreover, no statistically significant association was found in the bi-directional longitudinal analyses, although large magnitudes in risks were produced when diabetes was investigated as a risk factor for severe spinal pain. The presence of positive associations only in the cross-sectional analyses, in which confounders are partially controlled, suggests that type 2 diabetes and back pain could be linked by other shared common risk factors (e.g. genetics).

### Association of back pain and type 2 diabetes

Back pain and other musculoskeletal pain disorders are common among patients with diabetes [7, 8, 11, 33–35]. Despite this, there is a scarcity of well controlled studies that have attempted to disentangle this relationship. This may be due to back pain possibly being considered a trivial comorbidity compared with other major health problems associated with diabetes, such as heart disease or stroke [34]. Nevertheless, compelling evidence suggests that people who suffer from type 2 diabetes and back pain usually present with greater signs of poor general health (e.g. hypertension and dyslipidemia) and progress to worse outcomes such as increased pain severity [6, 34, 36, 37]. In agreement with previous studies, we found that type 2 diabetes was more strongly associated with severe cases of chronic spinal pain (adjusted OR 3.33, 95% CI 1.47 to 7.53), NP (adjusted OR 2.28, 95% CI 1.24 to 4.21), and LBP (adjusted OR 1.63, 95% CI 1.00 to 2.64).

Our cross-sectional results also indicated the association between diabetes and chronic neck and spinal pain tended to be more consistent, and often stronger, among females than in males. The underlying mechanisms for this difference is still unknown, but several reasons could be speculated. For example, sex hormones that are predominant in females (e.g. estrogen) can affect the immune system and increase the inflammatory response, which may result in a greater predisposition to develop diabetes and spine degeneration [38–43]. Higher prevalence of back pain and more rapid changes in spine degeneration have been reported in females after menopause [39, 41–45]. In our sample, most females were in their mid-life period (mean age: 54 years), which coincides with the timing of menopausal age [46]. As the majority of women with type 2 diabetes are older and frequently diagnosed during or after menopause [39], the understanding of underlying mechanisms of back pain and degeneration in this population may deserve further exploration.

### Causal relationship between type 2 diabetes and back pain

In light of the findings from the cross-sectional co-twin and the longitudinal analyses, the relationship between type 2 diabetes and back pain might not be as simple or direct as previously believed. After further adjustment for a large number of potential disease confounders, such as genetics and early environmental factors shared by twins, the significant associations between type 2 diabetes and back pain did not persist suggesting that a causal relationship between the two diseases is not likely. Our findings are consistent with results from two other twin studies that investigated lumbar spine degeneration in twin pairs discordant for diabetes status [24, 27]. One of these studies [24] investigated lumbar spine degeneration and bone density, using magnetic resonance imaging, among nine MZ twin pairs discordant for insulin-dependent diabetes. No difference was observed in lumbar disc degeneration or bone density scores between twins with or without a diagnosis of diabetes [24]. Likewise, a recent co-twin study including 33 MZ and DZ pairs discordant for type 2 diabetes reported that twins with type 2 diabetes did not present with higher lumbar degeneration scores than those without [27]. The findings from our study and these other co-twin studies [24, 27] indicate that a causal link between these health conditions are unlikely. Similarly, our longitudinal analyses based on two to four years follow-up showed that type 2 diabetes possibly does not increase the risk of back pain, or vice-versa, suggesting no temporal effect, one of the important indicators of a causal relationship between variables [47]. However, we acknowledge that the lack of significant associations in our co-twin and longitudinal analyses may be due to the small sample size, which may result in our analyses being underpowered to identify a relationship, if in fact, it exists. Some large magnitude risks were identified (e.g. diabetes as a risk factor for severe spinal pain, particularly in women), which might prove statistically significant if larger samples of twins were available.

### Implications of study findings for clinical practice and research

Although our results question a possible causal relationship between type 2 diabetes and back pain, this study suggests that LBP and NP are associated with type 2 diabetes. Our findings provide guidance for health professionals that people with both diabetes and back pain are more likely to present with more severe levels of pain compared to those without diabetes. Screening for back pain in patients with diabetes could be incorporated in clinical settings as an approach to avoid subsequent pain-related disability and minimize the progression of back pain and diabetes. At present, to our knowledge, only one study has investigated the efficacy of an intervention for this population, and found that osteopathic manual treatment resulted in a clinically relevant reduction of LBP severity over 12 weeks (medium effect size; Cohen d = 0.7) compared to sham treatment. Interestingly, better metabolic profile was also observed in the intervention group after treatment as serum concentration of TNF-α significantly reduced. TNF-α is considered a contributing factor for metabolic disturbances such as insulin resistance and dyslipidemia in type 2 diabetes [48]. Consequently, studies are needed to investigate interventions that can be delivered to patients suffering from both back pain and diabetes with the aim of minimizing diseases’ progression and their related complications.

### Strengths and limitations

The strengths of our study include the use of a co-twin design, allowing for within-twin pair comparisons naturally adjusted for genetics and other shared childhood factors such as diet and parental characteristics (e.g. socioeconomic status and lifestyle), that could affect both diabetes and back pain. In addition, we only included participants with a confirmed diagnosis of diabetes (via the Spanish Health Registry), which adds validity to the ascertainment of the presence of diabetes. An investigation of causality using longitudinal data accompanied the cross-sectional analyses, to assess whether there was a bi-directional relationship between type 2 diabetes and back pain.

There are also potential limitations that should be taken into account when interpreting our results. Firstly, our measure of back pain was relatively simplistic and did not include screening for back pain due to a specific cause (e.g. trauma or spinal deformity). This additional information could have contributed to a better understanding of the diabetes-back pain relationship, as cases of back pain due to a specific cause should have been excluded. Secondly, as expected due to the moderate back pain heritability [25, 26], there were few twin pairs discordant for back pain identified in our sample. As a result, it is possible that some co-twin analyses were underpowered. This issue seems to be more pronounced in the analyses for the outcome of ‘severe pain’ and when the analyses were stratified by sex. Despite the issue of power in some analysis, we believe our results because the matching generated by the co-twin design may have the potential to overcome this limitation [49, 50]. Furthermore, our findings were consistent with two other previously published co-twin studies that investigated the link between disc degeneration and diabetes [24, 27]. Inadequate power could have also affected the statistical significance and confidence intervals observed in our longitudinal analyses. Secondly, it is possible that the proposed biological mechanisms underlying the link between type 2 diabetes and back pain (e.g. spinal degeneration due to low-grade systemic inflammation) are slow and progressive. The maximum follow-up duration in the longitudinal analyses of this study was four years. As such, the temporal effect (time frame of a potential cause and effect) may need to be explored with longer follow-ups. Lastly, the generalizability of our results to global populations needs to be undertaken with caution. Our sample comprised older Spanish people from a Mediterranean region (Murcia) with a high prevalence of obesity and type 2 diabetes [51]. Therefore, before drawing definitive conclusions regarding causation, we emphasize the need for further prospective twin studies using a more heterogeneous and larger cohort with a more extended follow-up period.

In summary, chronic back pain (NP, LBP and both) was associated with the prevalence of type 2 diabetes. Stronger associations were observed for more severe cases of pain. Genes or environmental factors that influence both conditions should not be excluded as factors confounding these associations. Given the increasing global prevalence of back pain and diabetes, further studies are warranted to understand the mechanisms behind these associations, as well as the strategies to optimize management and healthcare utilization in this population.
